# COVID‐19 rapidly increases MDSCs and prolongs innate immune dysfunctions

**DOI:** 10.1002/eji.202249827

**Published:** 2022-06-24

**Authors:** Irene T. Schrijver, Charlotte Théroude, Nikolaos Antonakos, Jean Regina, Didier Le Roy, Pierre‐Alexandre Bart, Jean‐Daniel Chiche, Matthieu Perreau, Giuseppe Pantaleo, Thierry Calandra, Thierry Roger

**Affiliations:** ^1^ Service of Infectious Diseases Lausanne University Hospital and University of Lausanne Lausanne Switzerland; ^2^ Service of Internal Medicine Lausanne University Hospital and University of Lausanne Lausanne Switzerland; ^3^ Service of Adult Intensive Care Medicine Lausanne University Hospital and University of Lausanne Lausanne Switzerland; ^4^ Service of Immunology and Allergy Lausanne University Hospital and University of Lausanne Lausanne Switzerland

**Keywords:** COVID‐19, Cytokines, Dendritic cells, Monocytes, Myeloid‐derived suppressor cells

## Abstract

We used unsupervised immunophenotyping of blood leukocytes and measured cytokine production by innate immune cell exposed to LPS and R848. We show that COVID‐19 induces a rapid, transient upregulation of myeloid‐derived suppressor cells (MDSCs) accompanied by a rapid, sustained (up to 3 months) hyporesponsiveness of dendritic cells and monocytes. Blood MDSCs may represent biomarkers and targets for intervention strategies in COVID‐19 patients.

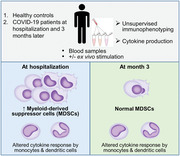

Inflammatory and danger signals stimulate hematopoiesis and the generation of myeloid‐derived suppressor cells (MDSCs) that suppress innate and adaptive immune responses [1]. High levels of blood MDSCs are associated with nosocomial infections, morbidity, and mortality in critically ill patients with sepsis [2]. Severe COVID‐19 is characterized by exuberant inflammation, leading to a cascade of immune‐related manifestations. Lymphopenia and impaired immune effector cell functions contribute to COVID‐19 pathogenesis and increase the risk of secondary infections and death [3]. While increased expression of MDSCs has been reported in COVID‐19 patients [4, 5, 6, 7], scare studies performed long‐term, longitudinal analyses in recovered patients.

We analyzed polymononuclear‐MDSCs (PMN‐MDSCs) and monocytic‐MDSCs (M‐MDSCs), the two main subgroups of MDSCs [1], in 56 COVID‐19 patients analyzed at hospitalization and 21 patients analyzed 3 months later. Patients with moderate (*n* = 45) and severe (*n* = 11, two died) COVID‐19 were similar for age, gender, underlying diseases, and history of immunosuppressive therapy. Severe COVID‐19 patients had higher leukocyte counts and longer hospital stay than moderate COVID‐19 patients (Supporting Information Table S1). Ten age‐ and sex‐matched healthy individuals were used as controls.

Blood samples were analyzed by flow cytometry and unsupervised clustering to quantify leukocyte subpopulations with an emphasis on PMN‐MDSCs and M‐MDSCs (Supporting Information Fig. S1; [8]). At study inclusion, patients expressed significantly less lineage positive (Lin^+^: CD3, CD7, CD19 or CD56 positive) cells; DCs; and classical, intermediate, and nonclassical monocytes than healthy controls, but fourfold more PMN‐MDSCs and twofold more M‐MDSCs (*p* = 0.03 and 0.01) (Fig. 1A). Interestingly, PMN‐MDSCs, M‐MDSCs and leukocytes counts were normal in patients (14 moderate and seven severe COVID‐19) analyzed 3 months after diagnosis.

**Figure 1:**
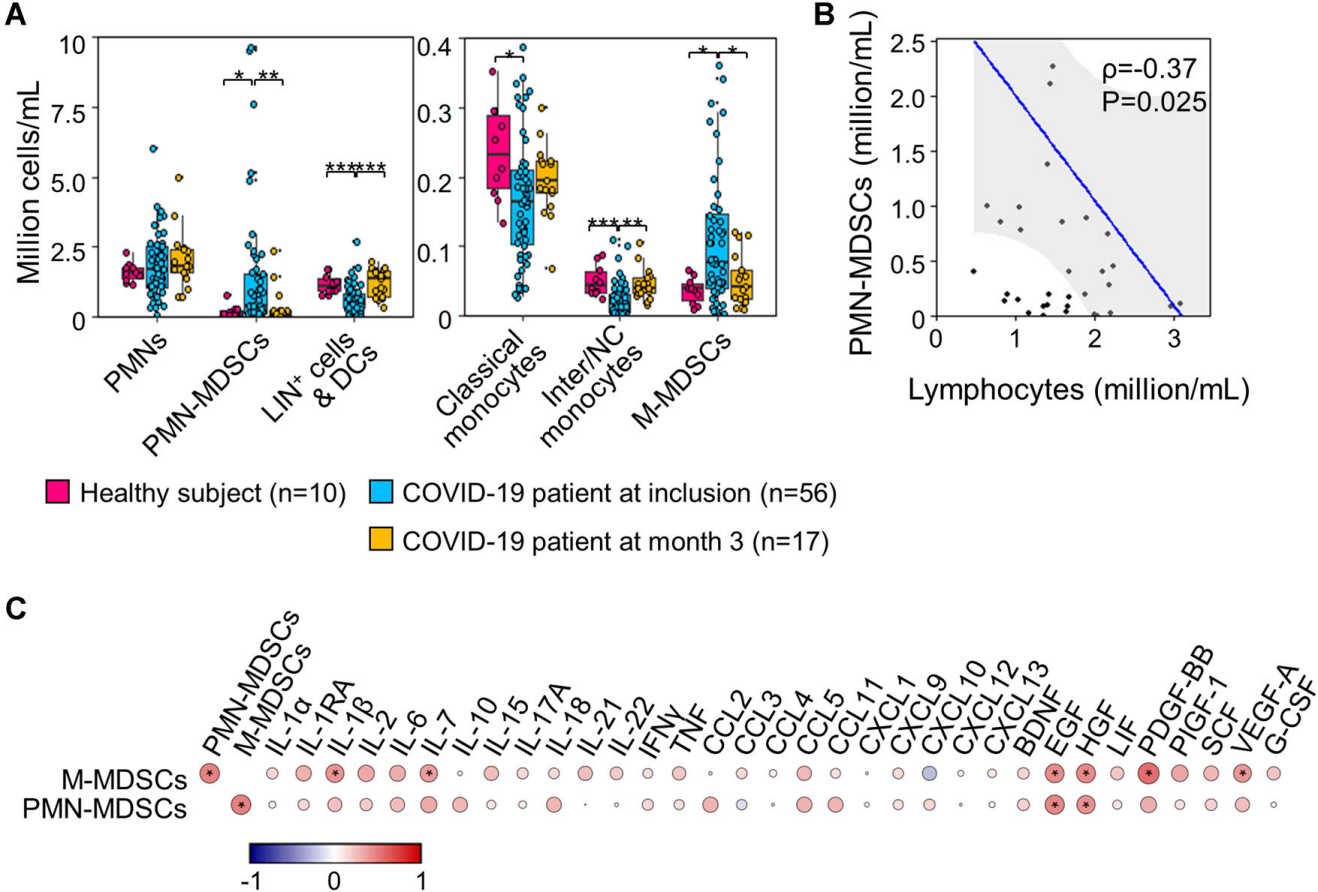
**MDSCs in COVID‐19 patients**. Blood was obtained from healthy subjects and COVID‐19 patients (45 moderate and 11 severe COVID‐19), and analyzed by flow cytometry and unsupervised clustering using FlowSOM. Serum mediators were quantified by multiplex bead assay. (**A**) Cell populations in healthy controls and COVID‐19 patients. Inter/NC monos: Intermediate/nonclassical monocytes, Lin: lineage, DCs: dendritic cells. Box plots show median, upper and lower quartiles. Whiskers show 5–95 percentiles. *p < 0.05, **p < 0.01, ***p < 0.001 (Mann–Whitney *U* test). (**B**) Scatter plot showing an inverse correlation between PMN‐MDSCs and lymphocytes at study inclusion. (**C**) Correlation plots of PMN‐MDSCs, M‐MDSCs, and serum mediators at study inclusion, calculated using Spearman's rank‐order correlation controlled for FDR; *p < 0.05.

At study inclusion, PMN‐MDSCs and M‐MDSCs counts were 10‐fold and fourfold higher in severe than in moderate COVID‐19 patients (Fig. 1A). Other cell populations were similar in severe and moderate COVID‐19 patients. PMN‐MDSCs and M‐MDSCs levels correlated with each other (*ρ* = 0.43; *p* = 0.03). PMN‐MDSCs inversely correlated with lymphocyte counts (Fig. 1B). A similar, not statistically significant, inverse correlation was detected between MDSCs and CD4^+^ and CD8^+^ T cells and T regulatory cells (Fig. S2). Since the levels of M‐MDSCs in blood, but not in the airways, correlated with COVID‐19 severity [5], the quantification of MDSCs in peripheral blood may represent an interesting biomarker of COVID‐19.

Thirty‐three cytokines/chemokines/growth factors (measured using a 49‐plex assay) were detected in the serum of patients (Fig. 1C). PMN‐MDSCs and M‐MDSCs correlated positively with most mediators (53/66 positive associations). Eight associations were statistically significant after correction for multiple testing. PMN‐MDSCs and M‐MDSCs correlated with epidermal growth factor (EGF; *ρ* = 0.47/0.44; *p* = 0.01/0.02) and hepatocyte growth factor (HGF; *ρ* = 0.42/0.46; *p* = 0.02/0.01). M‐MDSCs correlated with IL‐1β, IL‐7, PDGF‐BB, and vascular endothelial growth factor (*ρ* = 0.42, 0.38, 0.56, 0.40; *p* = 0.03, 0.05, <0.0001, 0.03) (Fig. 1C). Interestingly, EGF, HGF, PDGF‐BB, and VGEF have been shown to expand and chemo‐attract MDSCs, and IL‐1β and IL‐7 to stimulate myelopoiesis and sustain the expansion and T‐cell suppressing activity of MDSCs [1, 2]. Thus, the inflammatory milieu in COVID‐19 patients contains mediators that promote the generation and activity of MDSCs.

Whole blood was stimulated with LPS and R848. Intracellular cytokine staining followed by flow cytometry analysis was used to quantify the proportion of monocytes and DCs producing TNF and IL‐6 (Fig. 2). In healthy controls, 0.02/4.3% of monocytes produced TNF/IL‐6 at baseline, 24/17% in response to LPS, and 79/46% in response to R848, respectively. The percentage of blood monocytes producing TNF and IL‐6 in response to LPS and R848 was 1.3‐ to 4.9‐fold lower in COVID‐19 patients. The reduction was more striking in severe than in moderate COVID‐19 patients. The impaired response of monocytes persisted 3 months (Fig. 2Aand B). In healthy controls, 0.6% of DCs produced TNF/IL‐6 at baseline, 38/36% in response to LPS, and 68/58% in response to R848. TNF and IL‐6 positive DCs were 2.1‐ to 5.1‐fold lower in COVID‐19 patients, more impaired in severe than in moderate COVID‐19 patients. Impaired cytokine response persisted 3 months (Fig. 2C and D).

**Figure 2:**
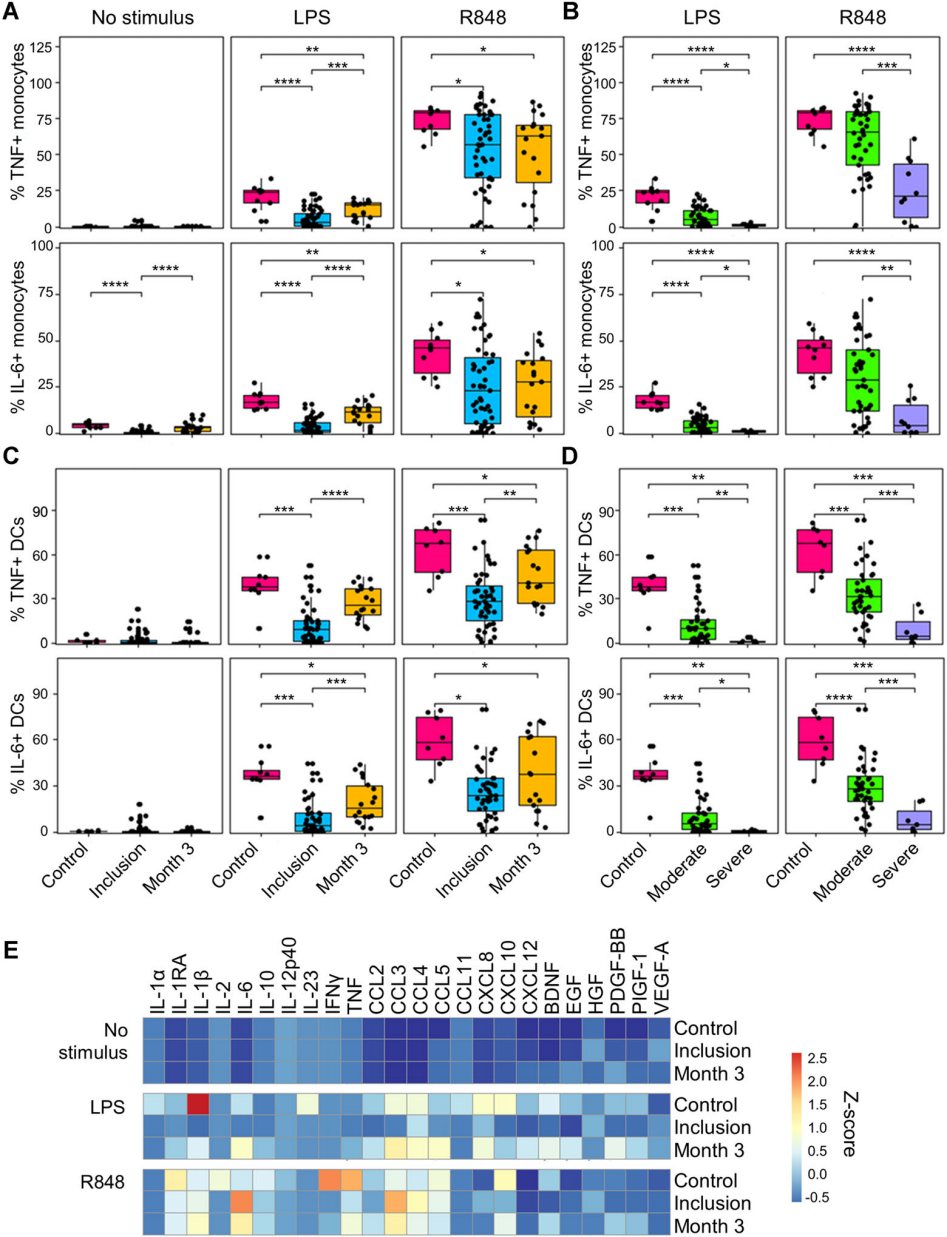
**Cytokine response by monocytes, DCs, and whole blood in COVID‐19 patients**. Blood was obtained from healthy controls and COVID‐19 patients. Blood was exposed for 4 h (**A–D**) or 24 h (**E**) to LPS (100 ng/mL) and R848 (5 μg/mL). (**A–D**) Cells were stained for intracellular cytokines and markers to identify monocytes and DCs, and analyzed by flow cytometry. Results are percentages of TNF^+^ and IL‐6^+^ cells within monocytes (**A and B**) and DCs (**C and D**). Box plots show median, upper and lower quartiles, whiskers 5–95 percentiles. *p < 0.05; **p < 0.01; ***p < 0.001; ****p < 0.0001 (Mann–Whitney *U* test). (**E**) Blood supernatants were used to quantify mediators by multiplex bead assay. Results are expressed as a heat map scaled expression plot based on *z*‐scores in healthy controls (*n* = 5) and COVID‐19 patients at inclusion (*n* = 13) and after 3 months (*n* = 12).

Upon stimulation with LPS and R848, 17 of 24 and 13 of 24 of cytokines were detected at lower concentrations in blood from patients than healthy controls (Fig. 2E). Interestingly, six of 24 and seven of 24 of the cytokines were detected at lower concentrations in patients analyzed after 3 months, implying prolonged immunological defects. Patients with moderate and severe COVID‐19 were similarly affected.

MDSCs represented 10%–15% of blood leukocytes, peaked in severe COVID‐19, and were associated with cytokine levels, lymphocytopenia, worse outcome, and impaired cytokine production by monocytes and DCs. Three months after inclusion, leukocyte counts were back to normal. Yet, blood, monocytes, and DCs still displayed reduced cytokine production, revealing long‐term immune disturbances. Likewise, MDSCs were normalized while cellular abnormalities were uncovered several weeks after SARS‐CoV‐2 infection [9]. Whether MDSCs play a role in persistent immune dysfunctions is unknown, but would involve long‐lasting imprinting independent from MDSCs elevated counts. The suppressive activity of MDSCs might vary over time, as reported during sepsis [2]. Overall, failure to restore immune homeostasis in COVID‐19 patients may be a driver of long‐COVID, increasing the risk of infections. Long COVID is reminiscent of the postsepsis syndrome characterized by immunosuppression associated with persistent low‐grade inflammation [10].

Our work has limitations. The number of patients was small, which may have limited the detection of differences or correlations. Additional markers could be used to trace MDSCs. However, we elected to minimize analytical variations by labeling whole blood quickly after drawing and analyzing flow cytometry data by unsupervised clustering. Finally, we have not assessed the immunosuppressive capacity of MDSCs. Yet, MDSCs of COVID‐19 patients were shown to inhibit the proliferation and cytokine production by T cells [4, 5, 6].

To conclude, our data suggest that MDSCs in peripheral blood represent biomarkers to stratify COVID‐19 patients. Targeting MDSCs and/or immune dysfunctions might proof useful to counterbalance immunosuppression, reduce nosocomial and long‐term infections, and decrease late mortality in severe COVID‐19 patients.

## Conflict of interest

The authors declare no commercial or financial conflict of interest.

### Peer review

The peer review history for this article is available at https://publons.com/publon/10.1002/eji.202249827.

## Supporting information

Supporting information.

## Data Availability

The data that support the findings of this study are available on request from the corresponding author. The data are not publicly available due to privacy or ethical restrictions.
