# Supplementary material for: COVID‐19 rapidly increases MDSCs and prolongs innate immune dysfunctions
Source: Eur J Immunol. 2022 Jun 24;52(10):1676–9. doi: 10.1002/eji.202249827 (PMC9350042; doi:10.1002/eji.202249827)
Supplement: Supplementary file 1 — Supporting information. [file EJI-52-1676-s001.pdf]

Supporting Information

Rapid increase of myeloid-derived suppressor cells and prolonged innate immune dysfunctions in patients with COVID-19

Irene T. Schrijver[1], Charlotte Théroude[1], Nikolaos Antonakos[1], Jean Regina, Didier Le Roy[1], Pierre-Alexandre Bart[2], Jean-Daniel Chiche[3], Matthieu Perreau[4], Giuseppe Pantaleo[4], Thierry Calandra[1], Thierry Roger[1]

[1]Service of Infectious Diseases, [2]Service of Internal Medicine, [3]Service of Adult Intensive Care Medicine and [4]Service of Immunology and Allergy, Lausanne University Hospital and University of Lausanne, Lausanne, Switzerland

Materials and methods

1. Subjects and ethic statement

Fifty-six hospitalized PCR-confirmed SARS-CoV-2 infected adult patients were enrolled in the Lausanne University Hospital (LUH) COVID-19 cohort study (Lausanne, Switzerland) during the time period of 01-04-2020 and 30-10-2020. The exclusion criterion for study enrolment was pregnancy. We did not exclude patients based on comorbidities including malignancies. Moderate COVID-19 was defined as hospital admission without the need for intubation, while severe COVID-19 was defined as hospital admission with mechanical ventilation for respiratory failure and/or death. Blood samples were collected at study inclusion. A second sample was collected 3 months later in 21 patients (14 moderate and 7 severe COVID-19). A control group comprised 10 age- and sex-matched healthy individuals. Exclusion criteria for healthy controls were prior diagnosis of SARS-CoV-2 infection, acute or chronic viral hepatitis, autoimmune disease, immunodeficiency and use of immunomodulatory drugs. The study was approved by the Commission cantonale d'éthique de la recherche sur l'être humain, Canton de Vaud,

Switzerland (CER-VD, Lausanne, Switzerland). Study participants provided written informed consent. Blood samples were treated fresh, in general within less than 1 hour.

2. Detection of MDSCs in whole blood by flow cytometry

One hundred microliter of EDTA-anticoagulated blood were incubated for 20 minutes at room temperature in the dark with a cocktail of antibodies directed against CD3, CD7, CD11b, CD14, CD15, CD16, CD19, CD33, CD45, CD56, CD135 and HLA-DR. Samples were diluted with 2 mL 1 x 1-step Fix/Lyse solution (eBioscience™, Thermo Fisher Scientific, Waltham, MA, USA), washed once with cell stain medium (CSM: PBS containing 0.5% BSA and 0.02% sodium azide) and acquired using an Attune NxT Flow Cytometer (Thermo Fisher Scientific). Debris, and doublets were excluded using manual gating (**Fig. S1A**), followed by FlowSOM unsupervised clustering using the biexponential transformed expression levels of CD11b, CD14, CD15, CD16, CD33, CD45, HLA-DR and lineage markers (CD3, CD7, CD19, CD56). Metaclustering was set on 30 populations manually merged into populations based on biological knowledge as represented in tSNE plots (**Fig. S1B**). PMN-MDSCs were identified based on their relatively low expression levels of CD16 and CD11b when compared to mature neutrophilic granulocytes and corresponded to $CD11b^{+}$ $CD14^{-}$ $CD15^{+}$ $CD16^{+}$ $CD33^{-}$ HLA-DR^{-} cells [1]. M-MDSCs were identified based on low expression levels of HLA-DR [1], and corresponded to $CD11b^{+}$ $CD14^{+}$ $CD15^{-/low}$ $CD16^{-}$ $CD33^{+}$ HLA-$DR^{-/low}$ cells (**Fig. S1C**). Reagents used for flow cytometry analyses are described in **Table S2**.

3. Blood cytokines, chemokines, growth factors and T cell populations

Serum concentrations of cytokines (IL-1α, IL-1RA, IL-1β, IL-2, IL-4, IL-5, IL-6, IL-7, IL-9, IL-10, IL-12p70, IL-13, IL-15, IL-17A, IL-18, IL-21, IL-22, IL-23, IL-27, IL-31, IFN-α, IFN-β, LIF, LT-α, TNF), chemokines (MCP-1/CCL2, MIP-1α/CCL3, MIP-1β/CCL4, RANTES/CCL5, Eotaxin-1/CCL11, GRO-α/CXCL1, IL-8/CXCL8, MIG/CXCL9, IP-10/CXCL10, SDF-1/CXCL12, BCA-1/CXCL13) and growth factors (NGF-β, BDNF, EGF, FGF-2, HGF, PDGF-BB, PIGF-1, SCF, VEGF-A, VEGF-D, BAFF, GM-CSF, G-CSF) were determined by multiplex bead assay using the Luminex xMAP Technology (Luminex Corporation, Austin, TX) and a BioPlex 200 array

reader (Bio-Rad Laboratories, Hercules, CA) as previously described [2]. Blood T cell populations were profiled by mass cytometry as thoroughly detailed in [2].

4. Whole blood stimulation assay

Three hundred µL EDTA-anticoagulated blood were incubated for 4 hours at 37°C with or without 100 ng/mL *Escherichia coli* O55:B5 ultrapure ultrapure lipopolysaccharide (LPS), or 5 µg/mL R848, 100 ng/mL. Brefeldin A (5 µg/mL, Invitrogen, Carlsbad, CA) was added at the beginning of incubation [3]. To analyze monocytic cells, 100 µL of reaction mixtures were incubated with LIVE/DEAD™ reagent and antibodies directed against CD14, CD16, CD19, CD33, CD56, HLA-DR and PD-L1/CD274. To analyze DCs, 200 µL of reaction mixtures were incubated with LIVE/DEAD™, Anti-Human Lineage Cocktail 2 (Lin-2, containing anti-CD3, CD14, CD19, CD20 and CD56 antibodies), and anti-CD1c, CD11c, CD16, CD123, HLA-DR and PD-L1/CD274 antibodies. After 20 minutes of incubation at room temperature in the dark, samples were diluted with 2 mL 1x 1-step Fix/Lyse Solution, washed with CSM, incubated for 10 minutes with CSM containing 0.3% saponin (Sigma-Aldrich, Saint Louis, MI), incubated for 20 minutes with CSM containing antibodies directed against TNF, IL-6 and IL-10, washed and acquired using an Attune NxT Flow Cytometer. Reagents are described in **Table S2**. Debris, doublets and dead cells (LIFE/DEAD^{TM}) were excluded by manual gating (**Fig. S1D**). SSC-A intermediate, CD33^{+}, CD3^{-} and CD20^{-} cells were selected by manual gating before applying FlowSOM unsupervised clustering (metacluster set at 20) based on the expression of HLA-DR, CD14, CD16, CD33, CD56 and SSC-A. A second round of clustering was applied to distinguish classical monocytes (HLA-DR^{+} CD14^{+} CD16^{-}), non-classical and intermediate monocytes (HLA-DR^{+} CD14$^{+/-}$ CD16^{+}), and M-MDSCs (HLA-DR$^{+/-}$ CD14^{+} CD16^{-}). To analyze DCs, HLA-DR^{+} and Lin2^{-} cells were selected by manual gating before applying FlowSOM (metacluster set at 12). Patients with < 30 DCs/mL were excluded. FlowSOM with metacluster set at 12 was applied to monocytes and DCs to analyze intracellular cytokine (data not shown). To quantify cytokine release by whole blood, 30 µL of EDTA-anticoagulated blood was incubated for 24 hours at 37°C with or without LPS, and R848 as described above.

Supernatants were used to quantify mediators by multiplex bead assay using the Luminex xMAP Technology.

5. Statistical analyses and softwares

Manual gating was performed with FlowJo™ Software version 10.6.2 (Ashland, OR). Statistical analyses and figure design were performed using R v.3.6.0 (R Foundation for Statistical Computing, Vienna, Austria). Baseline characteristic comparisons were made using Mann-Whitney U, Chi square or Kruskall-Wallis tests for skewed variables and student's t-test or Chi square for normal distributed variables. Cytokine and flow cytometry data were compared using the Kruskall-Wallis test, Mann-Whitney U, or Spearman's rank correlation controlling for False Discovery Rate (FDR) using the Benjamini and Hochberg step-up procedure. A 2-tailed $p<0.05$ was considered statistically significant.

Ethics

The study was approved by the Commission cantonale d'éthique de la recherche sur l'être humain, Canton de Vaud, Switzerland. We collected blood samples after written informed consent provided by study participants.

Author contributions

ITS, CT, and TR designed the study. PAB, JDC, MP, GP, TC and TR designed the LUH-COVID19 cohort. JR provided clinical characteristics of patients. ITS, CT, NA, DLR, and MP processed the samples. ITS analyzed raw data. All the authors interpreted the data. ITS and TR wrote the manuscript. All the authors revised the manuscript.

Data availability statement

The data that support the findings of this study are available from the corresponding author on reasonable request. Restrictions apply to due to privacy or ethical restrictions.

Supplementary Table 1. Patient's characteristics

Characteristic	Control	Moderate COVID-19	Severe COVID-19	3 months after study inclusion
Number of subjects	10	45	11	21
Gender, male	7 (70%)	31 (70%)	7 (64%)	15 (71%)
Age [years]	58 [55-65]	62 [53-74]	60 [48-63]	61 [54-75]
Charlson comorbidity index	-	3 [1.8-6]	2 [1-4.5]	2 [1-6]
Immunosuppressive drugs	-	7 [16%]	1 [5.3%]	1 [4.8%]
Days of symptoms before inclusion	-	7 [5-10]	7 [5-11]	9 [6.5-11]
Length of hospital stay[#]	-	3.5 [1.8-6.5]	24 [21-27]***	5 [2.5-17]
Death	-	-	2 (18%)	-
Leukocytes [x 10^9 cells/L]	3.4 [3.2-3.7]	3.0 [2.4-4.7]	7.0 [3.3-10.9]*	3.9 [3.2-4.8]
PMN-MDSCs [x 10^9 cells /L]	0.1 [0.07-0.2]	0.2 [0.08-1.0]	2.3 [0.6-8.6]**	0.1 [0.06-0.2]
M-MDSCs [x 10^9 cells /L]	0.04 [0.02-0.05]	0.05 [0.03-0.1]	0.22 [0.11-0.31]**	0.04 [0.02-0.07]

Data are n (%) or median [IQR]. [#]Excluding non-survivors, from moment of inclusion to hospital discharge. Statistics between moderate and severe COVID-19 patients: *p<0.05, **p<0.01, ***p<0.001.

Supplementary Table 2. Reagents

Antibodies and live/dead viable reagent used in flow cytometry

Target	Clone	Fluorochrome	Company	Reference
CD1c	L161	AF700	Biolegend	331530
CD11b	Bear1	PC-7	Beckman Coulter	A54822
CD11c	B-ly6	PE-TXR	BD Pharmingen	562393
CD123	6H6	BV711	Biolegend	306030
CD135	BV10A4H2	PE	Biolegend	313305
CD14	18D11	FITC	ImmunoTools	21620143
CD14	RMO52	APC-AF750	Beckman Coulter	B92421
CD15	80H5	Pacific Blue	Beckman Coulter	B49218
CD16	3G8	PB	BD Pharmingen	558122
CD16	3G8	ECD	Beckman Coulter	B49216
CD19	SJ25C1	APC-C7	BD Pharmingen	557791
CD19	J3.119	AlexaFluor 700	Beckman Coulter	B76284
CD274	MIH1	PE-Cy™7	BD Pharmingen	558017
CD3	SP34	APC-C7	BD Pharmingen	557757
CD33	WM33	BV711	BD Pharmingen	563171
CD33	D3HL60.251	APC	Beckman Coulter	IM2471
CD45	J33	Krome orange	Beckman Coulter	B36294
CD56	HCD56	AF700	Biolegend	318316
CD56	HCD56	AlexaFluor 700	Biolegend	318316
CD7	M-T701	AlexaFluor 700	BD	561603
HLA-DR	REA332	APC-Vio770	Miltenyi Biotec	130-104-871
HLA-DR	Immu-357	PE-TXR	Beckman Coulter	B94238
HLA-DR	Immu-357	FITC	Beckman Coulter	IM1638U
IL-10	JES3-9D7	PE	BD Pharmingen	559337
IL-6	MQ2-13A5	PerCP/Cy5.5	Biolegend	501117
Lin-2	Multiple	FITC	BD	643397
LIVE/DEAD™		Fixable Aqua	Invitrogen	L34957
TNF-α	MAb11	APC	Biolegend	307626

Other reagents

Name	Company	Reference
1-step Fix/Lyse Solution (10X)	eBioscience	00-5333-57
Bovine serum albumin	Sigma-Aldrich	A7906
Brefeldin A	Invitrogen	B7450
Escherichia coli O55:B5 ultrapure lipopolysaccharide	Invivogen	tlrl-pb5lps
R848	Invivogen	tlrl-r848-5
Saponin	Sigma-Aldrich	SAE0073-10G
Sodium azide	Sigma-Aldrich	71289

Supplementary Figures

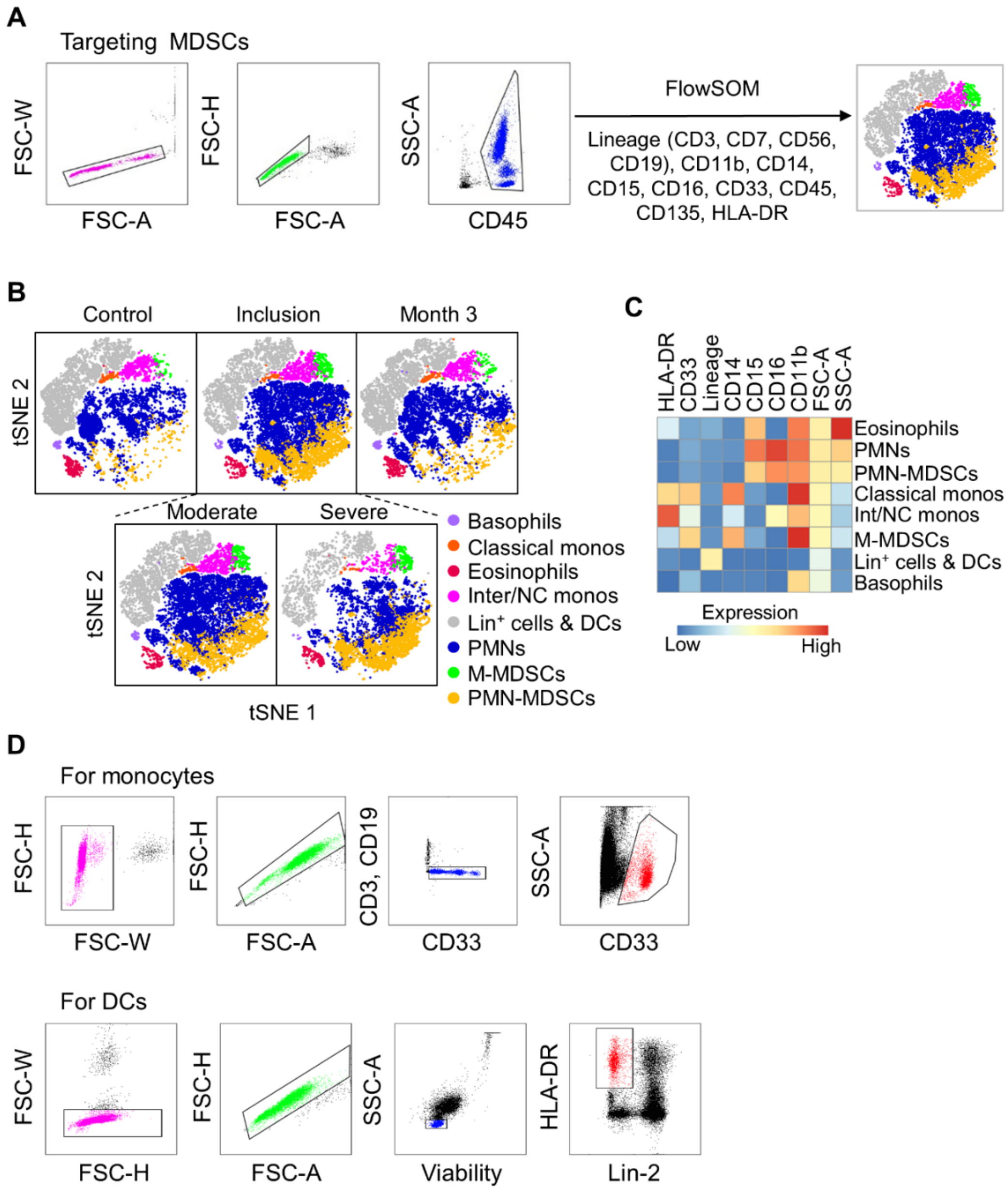

Supplementary Figure 1. Gating strategy and clustering analyses. Blood was obtained from 10 healthy subjects and 56 COVID-19 patients (45 with moderate COVID-19, 11 with severe COVID-19) at study inclusion and after 3 months (n=17) and analyzed by flow cytometry and, for MDSCs, unsupervised clustering using FlowSOM. (**A, D**) Gating strategy to exclude debris, doublets and non-hematopoietic cells to analyze blood MDSCs, monocytes and DCs by flow cytometry. (**B**) t-SNE plots of leukocyte populations. (**C**) Expression levels of cell surface markers and FSC-A/SSC-A of leukocyte populations.

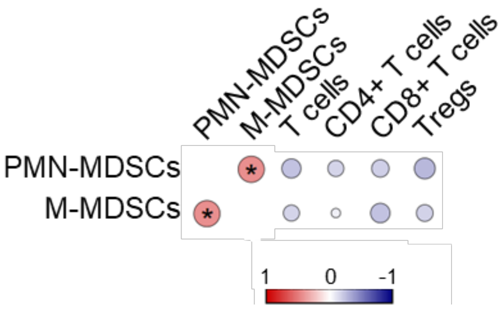

Supplementary Figure 2. Correlation plots of PMN-MDSCs, M-MDSCs, and lymphocyte populations (n=48). Correlations were calculated using Spearman's Rank-Order Correlation controlled for FDR. *$p<0.05$.

References

1. **Bronte, V., et al.**, Recommendations for myeloid-derived suppressor cell nomenclature and characterization standards. Nat Commun, 2016. 7:12150.
2. **Perreau, M., et al.**, The cytokines HGF and CXCL13 predict the severity and the mortality in COVID-19 patients. Nat Commun, 2021. 12:4888.
3. **Herderschee, J., et al.**, High-dimensional immune phenotyping of blood cells by mass cytometry in patients infected with hepatitis C virus. Clin Microbiol Infect, 2022. 28:611.e1-611.e7.
